# An epigenetic hypothesis for the genomic memory of pain

**DOI:** 10.3389/fncel.2015.00088

**Published:** 2015-03-24

**Authors:** Sebastian Alvarado, Maral Tajerian, Matthew Suderman, Ziv Machnes, Stephanie Pierfelice, Magali Millecamps, Laura S. Stone, Moshe Szyf

**Affiliations:** ^1^Department of Biology, Stanford UniversityPalo Alto, CA, USA; ^2^Department of Pharmacology and Therapeutics, Faculty of Medicine, McGill UniversityMontréal, QC, Canada; ^3^Sackler Program for Epigenetics and Developmental Psychobiology, McGill UniversityMontréal, QC, Canada; ^4^Department of Anesthesiology, Stanford UniversityPalo Alto, CA, USA; ^5^Integrated Program in Neuroscience, McGill UniversityMontréal, QC, Canada; ^6^Alan Edwards Centre for Research on Pain, McGill UniversityMontréal, QC, Canada; ^7^Faculty of Dentistry, McGill UniversityMontréal, QC, Canada; ^8^Department of Anesthesiology, Anesthesia Research Unit, Faculty of Medicine, McGill UniversityMontréal, QC, Canada

**Keywords:** chronic pain, epigenetics, neuropathy, prefrontal cortex, DNA methylation, synaptotagmin, neuroplasticity

## Abstract

Chronic pain is accompanied with long-term sensory, affective and cognitive disturbances. What are the mechanisms that mediate the long-term consequences of painful experiences and embed them in the genome? We hypothesize that alterations in DNA methylation, an enzymatic covalent modification of cytosine bases in DNA, serve as a “genomic” memory of pain in the adult cortex. DNA methylation is an epigenetic mechanism for long-term regulation of gene expression. Neuronal plasticity at the neuroanatomical, functional, morphological, physiological and molecular levels has been demonstrated throughout the neuroaxis in response to persistent pain, including in the adult prefrontal cortex (PFC). We have previously reported widespread changes in gene expression and DNA methylation in the PFC many months following peripheral nerve injury. In support of this hypothesis, we show here that up-regulation of a gene involved with synaptic function, *Synaptotagmin* II (*syt2*), in the PFC in a chronic pain model is associated with long-term changes in DNA methylation. The challenges of understanding the contributions of epigenetic mechanisms such as DNA methylation within the PFC to pain chronicity and their therapeutic implications are discussed.

## Chronic Pain is Associated with Anatomical, Morphological, and Physiological Changes in the Adult Prefrontal Cortex

Chronic pain is associated with a multitude of co-morbidities, including depression, anxiety, cognitive impairment, memory deficits and loss of motivation both in humans (Sharp and Keefe, 2005) and in animal models (Low, 2013; Schwartz et al., 2014; Tajerian et al., 2014). Rather than the pain itself, these higher-order functions, mediated by supra-spinal structures, can have the biggest impact on quality of life in chronic pain patients (Nicholson and Verma, 2004).

Chronic pain changes brain anatomy and function. Studies in rodent models of chronic pain have demonstrated pain-related modifications in areas including the hippocampus, amygdala, perirhinal cortex, and prefrontal cortex (PFC; Seminowicz et al., 2009; Mutso et al., 2012; Alvarado et al., 2013; Tajerian et al., 2013, 2014). These findings extend to humans—multiple studies have reported decreased gray matter, reduced cortical thickness, abnormal cortical function, and altered connectivity in various brain regions in a wide range of chronic pain conditions including low back pain (Giesecke et al., 2004; Apkarian et al., 2005; Schmidt-Wilcke et al., 2006; Tagliazucchi et al., 2010; Berger et al., 2014), headache (Schmidt-Wilcke et al., 2005), fibromyalgia (Kuchinad et al., 2007; Schmidt-Wilcke et al., 2007), post-stroke pain (Krause et al., 2014), complex regional pain syndrome (Pleger et al., 2014), burning mouth syndrome (Khan et al., 2014), and irritable bowel syndrome (Davis et al., 2008). The magnitude of these changes has been related to the duration and the intensity of chronic pain (Apkarian et al., 2004).

While changes in some brain regions are associated with specific chronic pain conditions, most studies report changes in common areas involved in pain modulation, including the PFC (Apkarian et al., 2009; Neugebauer et al., 2009). Interestingly, the PFC has been implicated in depression, anxiety and cognitive impairment, all of which are frequently associated with chronic pain. Pain-related pathological changes in the PFC may therefore contribute to the emergence of emotional and cognitive impairments.

In order to determine if chronic pain could induce pain-related changes in brain anatomy, Seminowicz et al. conducted a longitudinal study of chronic neuropathic pain in rats (Seminowicz et al., 2009). Consistent with the human literature, pain-related decreases in frontal cortex volume were observed in rats subjected to peripheral nerve injury as adults. These changes were not observed until approximately 4 months post-injury and were temporally correlated with the development of anxiety-like symptoms in the same animals. Thus, pain-related changes in the PFC are a consequence of chronic, but not acute pain.

At the molecular/cellular level, animal studies have demonstrated neuropathy-induced altered dendritic branching and spine density. For example, basal dendrites had longer branches in the PFC in animals with peripheral nerve injury than in controls (Metz et al., 2009). Changes in neuroanatomy are also linked to functional differences within the firing of pyramidal neurons (Centeno et al., 2009; Metz et al., 2009) and reduced connectivity between the PFC and other brain regions (Cardoso-Cruz et al., 2013a,b).

Ongoing chronic pain not only induces changes in the PFC but also actively maintains them. We have shown that pathological changes in the PFC in individuals with chronic low back pain (cLBP) can be reversed with effective pain management (Seminowicz et al., 2011). Specifically, cLBP-associated cortical thinning in the dorsolateral PFC (DLPFC) was reversed post-treatment, and the magnitude of this reversal correlated with the reduction of both pain and physical disability. Furthermore, abnormal activity in the DLPFC during an attention-demanding cognitive task in cLBP patients was reduced towards normal levels following treatment. The ability of the adult PFC to undergo neuroplastic changes is further supported by studies in healthy subjects (Hötting and Röder, 2013). For example, increased physical activity resulted in improved memory and increased local gray matter volume in the PFC in adult volunteers (Ruscheweyh et al., 2011).

These data indicate that long-term structural and functional brain abnormalities—specifically in the PFC—are induced by chronic pain. Furthermore, they suggest that treating chronic pain can restore normal brain function in the adult human PFC and raise the possibility that therapies targeting pathological changes in the PFC have therapeutic utility. Finally, the reversibility suggests that these changes are unlikely to be due to neurotoxicity; in contrast, the underlying mechanisms must be both long-lasting and reversible.

## Pain-Related Changes in Gene Expression in the Adult Prefrontal Cortex

Given the extensive structural and functional abnormalities in the PFC associated with pain persistent changes in genomic programming are likely to contribute to both chronic pain and to the associated co-morbidities. For example, in a model of acute facial pain, genes related to immune function and neutrophil activation are over-expressed in the PFC (Poh et al., 2012). In chronic neuropathic pain, we observed differential RNA expression of 1147 genes. Some of these genes are associated with functional pathways involved in neuronal development, cell differentiation and growth in the PFC 6 months following peripheral nerve injury (Alvarado et al., 2013). Furthermore, the majority of these differentially expressed transcripts were enriched for narrow, broad and bivalent types of promoters (Table 1, Lenhard et al., 2012). That is, narrow and weak promoters represent tissue specific and general cell cycle processes, respectively. This suggests that transcriptional landscape is accompanied with changes that are enriched for tissue-specific changes than those involved with the general cell cycle. Given the scope of long-term anatomical changes and the large number of differentially expressed transcripts, the transcriptional machinery itself is likely to become dysregulated as chronic pain progresses. Within upregulated transcriptional networks, we identified pathways that were relevant to cellular growth, differentiation, structural function and neuronal function (Alvarado et al., 2013). This is important since pain-related structural and functional changes may involve several of these pathways in degenerative/regenerative changes in the cortex due to altered neuronal/dendritic architecture (Freeman et al., 2008), glial loss or modifications to the extracellular environment (Tajerian and Clark, 2015).

**Table 1:** **Classification of transcriptome-identified promoters**.

Gene type	Number of genes*	Differentially expressed*	Enrichment *p*-value**	Enrichment status^+^
Housekeeping	3292	29	1.60E-13	Depleted
Weak promoter	5262	118	0.07	Depleted
Narrow promoter	930	43	1.20E-04	Enriched
Broad promoter	1279	51	9.50E-04	Enriched
Bivalent	3400	166	4.90E-18	Enriched

*Considers only protein coding genes. **Enrichment was tested by Fisher’s exact test. ^+^Depleted/enriched identify cases where the number of genes is smaller/larger than expected.

Within the differentially expressed transcripts associated with nerve injury, one third were annotated protein-coding transcripts, and the remaining were an assortment of non-coding RNAs of various identities (lincRNAs, miRNAs, etc.). These data offers additional insight into the function of previously uncharacterized transcripts that do not code for protein. Thus, non-coding RNAs within the PFC may also play a role in neuropathic pain. Our transcriptome analysis also revealed enrichment of non-annotated transcripts derived from within and outside of gene bodies (non-annotated in mm9 genome assembly) (Alvarado et al., 2013). While little is known regarding their function, reports suggest that non-coding RNAs within specific cell types and neuroanatomical structures may be artifacts of chromatin remodeling (Mercer et al., 2008). Specific examples of non-coding RNAs have also been shown to mediate antisense repression in primary afferent neurons (Zhao et al., 2013) and to act as epigenetic regulators in the nucleus accumbens in neuropathy (Imai et al., 2011). Given their diverse functions and origins, more studies and deeper sequencing are required to reveal a causal role of non-coding RNAs to chronic pain (Stefani and Slack, 2008; Mercer et al., 2009).

A growing body of literature using sequencing technology has revealed transcriptome signatures in illnesses that are co-morbid with chronic pain in the PFC, including depression (Sibille et al., 2004), sleep disorders (Maret et al., 2007), anxiety (Sibille et al., 2004; Virok et al., 2011), and cognitive impairment (Wood et al., 2013; Humphries and Kohli, 2014). While geneticists have long sought a heritable mutational basis for disease susceptibility, genome-wide association studies and the search for such genes have found few risk alleles that account for these phenomena in a broader population (Kraft and Hunter, 2009). In humans, the temporal transcriptomic architecture of the PFC remains consistent despite the extensive genetic variability existing in natural human populations (Colantuoni et al., 2011). Thus, factors that regulate transcriptional changes from DNA lie “above” genetic determinants.

## Epigenetic Mechanisms and Function in the Prefrontal Cortex

We propose epigenetic modulation of gene expression as a mechanism contributing to long-term plasticity in the nervous system in general and in the PFC specifically in chronic pain conditions. Epigenetics is a broad term used to describe modifications to the function of a gene that do not alter the sequence of a gene itself. In the adult brain, this definition encompasses stable changes to gene function beyond those associated with cellular differentiation following somatic cell division. Epigenetic mechanisms involve transcriptional regulation through either chromatin modification or through the covalent modification of the DNA molecule itself. The addition of a methyl group to the 5’ position of the cytosine ring, catalyzed by DNA methyl transferase, DNMT1, has been reported to interact with transcripts and inhibit the methylation of particular genomic loci (Di Ruscio et al., 2013). This is particularly interesting given the abundance of non-coding RNAs within the brain of injured animals. Furthermore, the methyl moiety of methylated DNA can be further modified by hydroxylation (Kriaucionis and Heintz, 2009) and carboxylation catalyzed by TET enzymes (Ito et al., 2010). More recently, the term “epigenetics” has begun to incorporate other mechanisms of regulation that function through higher order chromatin folding (Cremer and Cremer, 2010), non-coding RNAs (Flanagan and Wild, 2007) and editing of mRNAs. Here we will focus primarily on DNA methylation as a molecular medium for storing broad, long-term changes in transcription in the PFC in chronic pain conditions.

Genomic methylation can be distributed within CpG dinucleotides (Bird, 1986) and non-CpG elements (Ramsahoye et al., 2000). In the adult mouse brain, 75% of this methylation occurs within CpG nucleotides (Guo et al., 2013) and in most tissues, methylation is stable, with only ~6% variation in differential methylation between tissues (Lister et al., 2009; Hon et al., 2013; Ziller et al., 2013). Differential methylation that occurs within these 6% cytosines can regulate transcription and splicing through multiple mechanisms. By regulating which genes are and are not expressed in an individual cell, DNA methylation allows the same genomic DNA to encode the multitude of phenotypes in multicellular organisms which emerge with cellular differentiation, such as the difference between brain cells and skin cells within the same individual (Razin and Riggs, 1980). For example, increased DNA methylation in promoters or enhancers silences their activity through the recruitment of transcriptional repressors and/or steric hindrance of methyl groups (Stein et al., 1982; Comb and Goodman, 1990). In contrast, increases in DNA methylation within gene bodies are associated with actively transcribing genes (Lister et al., 2009; Hon et al., 2013). However, it is unclear whether the methylation in gene bodies plays a role in regulation of gene expression as most of the studies documenting this are descriptive and there is no clear mechanism that links gene body methylation and either transcription initiation or elongation. It is clear that the issue of “gene body methylation” requires better biochemistry which has been completely lacking in the recent flurry of genome wide mapping studies. Additionally, DNA methylation has been implicated in regulating alternative splicing in mammals (Shukla et al., 2011) and invertebrates (Foret et al., 2012) by pausing transcriptional machinery within stretches of methylated DNA. Perhaps the only strong evidence to date on the role of DNA methylation in controlling gene expression comes from the pioneering studies of Doerfler (Vardimon et al., 1982) and Razin and Cedar (Stein et al., 1982) from the early 80 s which showed that promoter methylation completely silences gene expression of transfected viral and genomic promoters. These studies have been repeated numerous times and, without exception, methylation of promoters silences gene expression. However, the mechanisms have been more difficult to understand. There is paucity of biochemical data that truly examines mechanisms of promoter silencing. The strongest data comes from pioneering studies in early nineties showing that binding of transcription factor is inhibited by methylation of the recognition element g (Comb and Goodman, 1990). In addition to this simple and attractive mechanism, a chromatin-based mechanism was proposed in the late nineties. Bird has suggested that methylated DNA binding proteins (MBDs; Nan et al., 1998) recruit complexes such as histone deacetylases that result in an inactive chromatin structure. However, later data (Baubec et al., 2013) shed some doubt on this simplistic understanding of the role of MBDs as they were found to bind both methylated and unmethylated genes as well as active and inactive genes. Although MeCP2 was proposed to be a ubiquitous suppressor of methylated promoters, analysis of gene expression in MeCP2 knock outs revealed silencing of 85% of transcripts whose expression was changed and conversely overexpression of MeCp2 resulted in induction of most genes that were altered by MeCP2 overexpression (Chahrour et al., 2008).

## Role of DNA Methylation in the Brain: Learning, Memory and Neurodegenerative Disease

In the brain, DNA methylation is a dynamic process throughout the entire life cycle. For example, in the developing mammalian brain, methylomes of neurons are widely reconfigured during synaptogenesis (Lister et al., 2013). In adults, DNA methylation is involved in memory and synaptic plasticity (Miller and Sweatt, 2007; Day and Sweatt, 2010) possibly through the regulation of DNMT3A/B in the forebrain (Feng et al., 2010). These patterns of DNA methylation have elucidated several molecular signatures underlying mental illness related to maternal deprivation (Weaver et al., 2004a; Massart et al., 2014b), depression (McGowan et al., 2009) and sleep disorders (Massart et al., 2014a).

A causal role between DNA methylation in the brain and learning and memory is supported by data from contextual fear learning paradigms showing, for example, aberrant and reversible methylation of calcineurin and brain derived neurotropic factor, both known regulators of synaptic plasticity (Lubin et al., 2008; Miller et al., 2010). In addition, the causal role of DNA methylating machinery (DNMTs) and their pharmacological inhibition have been directly linked to several additional learning paradigms (Miller and Sweatt, 2007; Miller et al., 2008; Feng et al., 2010; Day et al., 2013). The role of DNA methylation within the brain has been further extended to the transduction of epigenetic marks into the germ line and the transmission of heritable traits related to olfactory behaviors (Dias and Ressler, 2014). Finally, the role of DNA methylation in the brain has been extended to pathological mental health conditions. For instance, in a recent epigenetic-wide association study in human autopsied brains, the methylation status of several CpGs was significantly associated with the burden of Alzheimer’s pathology (De Jager et al., 2014). In schizophrenia, DNA methylation profiles from the frontal cortex have shown aberrant downstream methylation of candidate regulators of disease pathology (Numata et al., 2014; Walton et al., 2014).

Hypothesis: Alterations in DNA Methylation in the Prefrontal Cortex Following Nerve Injury Mediate the Long-Term Genomic Impact of Pain as a Fundamental Mechanism Contributing to the Chronicity of Pain. This Mechanism Raises the Potential of Dna Methylation-Modulating Therapy for Reversing Chronic Pain.

**Figure 1:**
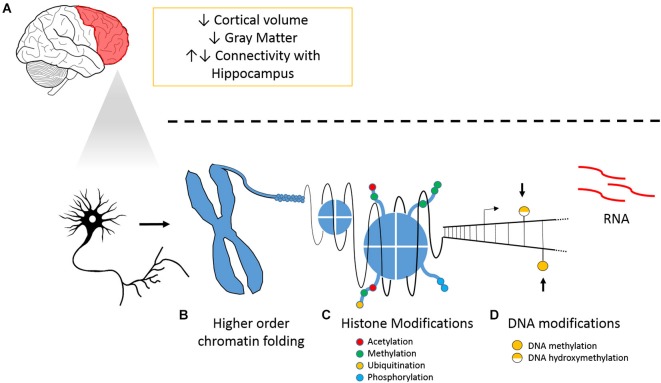
**Chronic pain and its tissue specific and cellular effects on the PFC. (A)** Overview of neuroanatomical and molecular changes that accompany pain and illustrations of **(B)** higher order chromatin folding, **(C)** histone modification; and **(D)** DNA modifications.

Our hypothesis underlines the importance of epigenetic regulation of PFC plasticity in chronic pain (Figure 1). We have previously reported hypomethylation in the PFC and amygdala 6 months following peripheral nerve injury (Tajerian et al., 2013). The amygdala is implicated in fear and anxiety, modulates the PFC and is modulated by it. Thus, we hypothesize that chronic pain leaves lasting changes in DNA methylation that stably alter genomic programming and could be responsible for pain chronicity and pain-associated co-morbidities. We have also previously shown that housing mice in environments with social and physical enrichment attenuated nerve injury-related changes in genomic hypomethylation within the PFC and reduced pain-like behaviors. We therefore also hypothesize that pain-related changes in methylation within the PFC are dynamic. While the changes in global DNA methylation demonstrate a shift in the DNA modification landscape related to pain-associated brain plasticity, the impact of altered DNA methylation on transcriptional regulation of individual genes remains to be clarified.

## Synaptotagmin II: A Proof-of-Principle Case Study

**Figure 2:**
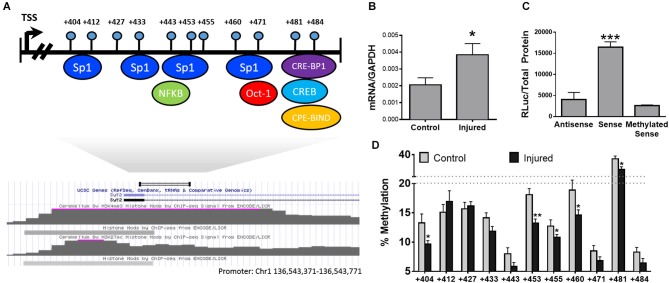
**DNA methylation and transcriptional regulation of the Syt2 Promoter. (A)** Physical map of the *syt2* regulatory region with predicted transcription factor binding sites below. **(B)** Transcription of *Syt2* in control and injured animals *n* = 6/group. **(C)** Luciferase promoter assay of the *syt2* promoter cloned into pCpGl in antisense, sense, and methylated sense directions. Transcription in the sense direction is blocked by methylation of the syn2 promoter *n* = 3/group. **(D)** Bisulfite methylation mapping of the putative *syt2* promoter upstream of the *syt2* transcriptional start site (TSS) demonstrating neuropathy-induced decreases in DNA methylation at multiple sites * = *p* < 0.05, ** = *p* < 0.005, *** = *p* < 0.001. Error bars = SEM.

Our hypothesis predicts that changes in DNA methylation in promoters and enhancers of individual genes will result in alterations in gene expression contributing to pain-related changes in brain structure and function. In order to test our hypothesis, we selected S*ynaptotagmin II* (*syt2*) [GenBank: AL596207.10] as our test case. *Syt2*, a known regulator of synaptic function, belongs to a family of membrane-trafficking proteins, is involved in synaptic vesicle docking and fusion, and acts as a calcium sensor for fast neurotransmitter release. Since *syt2* was previously shown to be upregulated in the PFC of neuropathic mice 6 months after injury (Alvarado et al., 2013), whether this dysregulation is under epigenetic control was investigated. To identify the 5’ regulatory regions of the gene we utilized previously deciphered whole epigenome map of regulatory regions in the mouse genome (ENCODE Project Consortium, 2011) to identify areas of the gene with promoter-like activity. A H3K4me3 peak (a histone modification which is a hallmark of promoters) (ENCODE Project Consortium, 2011) was located downstream of the transcription start site of the gene (see map in Figure 2) and the state of methylation of this region was mapped in the PFC in nerve injured and control mice.

Pyrosequencing analysis of the methylation state of 11 CpG sites residing downstream of the *syt2* transcriptional start site (TSS; Figure 2A) showed significant hypomethylation in the nerve injured group compared to controls (Figure 2B, *n* = 6/group). In order to directly determine whether methylation of CG sites in this *syt2* regulatory region affects the ability of this region to activate transcription, the region was subcloned into a CpG-deficient luciferase reporter; the same region was subcloned in the antisense direction as a negative control. The promoter was then methylated *in vitro* using Sss1 DNA methyltransferase or mock methylation was performed in absence of enzyme as a negative control. Since the vector does not contain CG sites, only CG sites in the inserted *syt2* region could be methylated *in vitro*. The mock methylated and methylated *syt2* luciferase reporters were transiently transfected into HEK293 cells. The luciferase assay results demonstrated that this *syt2* region directs transcriptional activity that is inhibited by the same methylation of CG sites that are differentially methylated *in vivo* in response to nerve injury (Figure 2C, *n* = 3/group). Interestingly, we observe changes in DNA methylation in a very limited area in the promoter. This is consistent with the hypothesis that DNA methylation silences promoter activity through interference with binding of factors discussed above. For example, we have shown previously that epigenetic programming by maternal care of the Glucocorticoid receptor gene involves site specific changes in one CG which is also the binding site for the transcription factor NGFI-A (Weaver et al., 2004a). We showed that methylation of the NGFI-A binding site inhibits NGFI-A binding and silences promoter activity (Weaver et al., 2007). Further, experiments are required to identify the transcription factor that is inhibited by DNA methylation in the *syt2* promoter.

Examination of syt2 methylation in animals that were subjected to nerve injury provides evidence in support of the hypothesis that DNA methylation regulates gene expression within the PFC in chronic pain. These changes were observed in the brain long after the original peripheral injury. Thus, our data provides a proof of principle for DNA methylation of regulatory regions as a mechanism for long-term reprogramming of gene expression in response to peripheral nerve injury. Further experiments are required to determine the full importance of this mechanism in reprogramming gene expression in the central nervous system following a chronic pain-producing injury.

*Syt2* expression is critical for synaptic transmission in caudal and forebrain regions (Pang et al., 2006; Kochubey and Schneggenburger, 2011). In general, synaptotagmin is a marker of synaptic density and plasticity and its increase is correlated with synaptogenesis (Masliah and Terry, 1993). Our data are consistent with previous findings showing increased *syt2* expression in the PFC of rats subjected to chronic restraint stress (Virok et al., 2011), supporting the hypothesis that chronic anxiety/stress (regardless if its source being chronic restraint or chronic pain) could result in profound changes in synaptic structure and/or function.

## Epigenetics of Chronic Pain: Current Understanding and Future Directions

While the role of DNA methylation in regulating transcription has been extensively validated, we are only beginning to understand its full effects on a genomic landscape. For example, DNA methylation within the PFC following nerve injury decreased by 12%, accounting for ~180,000 CpG sites across the genome (Tajerian et al., 2013). A 12% decrease in methylation relating directly to transcripts within the genome would reflect a dramatic effect on the transcriptome. However, we only observed a change of 800 protein coding transcripts considering >1.5% of our genome codes for protein (Lander et al., 2001). Furthermore this observation does not account for cytosine methylation that occurs outside of a CpG dinucleotide that has been considered to be more widely distributed within the brain (Lister et al., 2013). This supports additional roles for DNA methylation and their relation to chromosomal integrity (Eden et al., 2003) and higher order chromatin structure (Jimenez-Useche et al., 2013). For example, telomeric and centromeric repeats are CpG-dense and heavily methylated in post-mitotic cells (Yoder et al., 1997; Han et al., 2008). Increased methylation at these sites would involve condensation of chromatin and increased stability of higher order structures. We speculate that such hypomethylation would also implicate repetitive elements within the genome such as SINE/LINE transposable elements, also shown to affect nuclear structure by defining chromosomal breaking points as seen in immunodeficiency disorders (Tuck-Muller et al., 2000). Our promoter analysis (Table 1) and differential transcriptome, shows a depletion of weak promoters (CpG island rich). This suggests that the protein-coding component of our data may rely on the DNA methylation of CpG island poor promoters (narrow- and broad-type promoters). As a result this extends our interpretation of genomic hypermethylation outside of the scope of protein coding genes. Our interpretation of genomic hypomethylation is limited by our approach using a restriction enzyme-coupled assay and not a comprehensive view of which genomic areas become hypomethylated in pain. Future studies using approaches such as methylated DNA immunoprecipitation and sequencing will offer the necessary resolution needed to reveal the nature of DNA methylation associated with pain.

The data collected thus far is limited to a single time point 6 months post-injury. It will be important to gain a temporal understanding of pain-related changes in DNA methylation. Future studies should examine that time course of changes within the brain. Are there wide-spread changes in methylation throughout the CNS one day after an injury? One month? Three months? Exactly what genes are dysregulated at each of these time points? Do these changes precede the development of cognitive or emotional changes or vice versa?

Given the magnitude of the pain-related changes in global methylation, it was surprising that no changes were observed in any of the known regulators of methylation (i.e., DNMTs, methylated DNA binding proteins) in our transcriptomic screen of the PFC. Is this because massive dysregulation of hypermethylation/hypomethylation through the DMNTs occurs early after injury but by 6 months has reached a new steady state? Given the chronicity of pain and its co-morbidities, we believe that the processes underlying neuroplasticity are part of the etiology of the disease and exist in a continuum of cause and effect. For example, while co-morbidities of chronic pain may not manifest themselves early following injury, the emergence of co-morbidities may initiate a cascade of transcriptional changes in the brain that program long-term changes. This cascade, modulated by DNA methylation and other epigenetic processes over the course of months to years, may feed into the pathology of chronic pain, thus increasing the burden of the disease. Would that imply that pain-related changes in methylation are more difficult to reverse months vs. days after injury? All of these questions merit further exploration. However, it should be noted that DNA methylation states of genes are regulated by many factors in addition to global levels of enzyme such as local presence of transcription factors and chromatin states of genomic loci, these rather than global changes in enzyme levels might be the determining causal agents of changes in DNA methylation in chronic pain.

Interpretation of changes of DNA methylation in the brain, as compared to other tissues, is complicated by cell heterogeneity. In the brain, it is difficult to attribute altered patterns of methylation to specific cell types. In Lister et al., mapping of the brain methylome, cell sorting enabled distinction between neurons compared to astrocytes (Lister et al., 2013). However, there is still extensive heterogeneity of neuronal cell types even within a single brain region. This is particularly important given that neurons within the PFC have more inter-individual variability in DNA methylation than do other cell types within the same tissue (Iwamoto et al., 2011). The difficulties in interpreting these changes are obvious when one considers a hypothetical brain region where there is 0% methylation at all glia-specific alleles and 100% methylation at all neuronal specific alleles, resulting in an observation that ~50% methylation. Future studies examining the contributions of different neuronal and non-neuronal cells using either cell sorting or histological methods are needed to begin to unravel these complexities.

It is important to note that epigenetic changes are not independent of one another. A great deal of crosstalk between DNA methylation has been reported (D’Alessio et al., 2007; Ou et al., 2007; Brinkman et al., 2012). It is therefore important to examine additional layers of epigenetic regulation. This additional tier of regulation may hold clues into the mechanisms of chronicity, specifically regarding the expression of specific gene families.

Finally, while this manuscript uses the example of syn2 to illustrate the main hypothesis, the role of individual genes or gene families is virtually unexplored. While some differentially methylated sites may be critically important in pain-related neuroplasticity, it is likely that many are not. Understanding epigenetic regulation at both the genome-wide and the gene-specific levels will provide insights into how chronic pain changes the brain and how to reverse it.

## Epigenetics of Chronic Pain: Potential Therapeutic Targets

One fundamental difference between epigenetic regulation and genetic polymorphisms is the potential of the former to be modulated by pharmacological manipulations (Szyf, 1994, 2009). In rodents, the behavioral impact of early life experience (i.e., maternal care) is reversible in the adult offspring with epigenetic drugs (Weaver et al., 2004a,b, 2005, 2006). The potential therapeutic value of interventions targeting epigenetic mechanisms underlying chronic pain has exciting clinical significance.

To date, most pharmacological interventions directly targeting epigenetic mechanisms have been used to provide evidence of epigenetic involvement rather than to explore viable treatment options. However, with the increased emphasis on pain epigenetics, it is plausible that more targeted approaches could be applied to the treatment of pain and its related comorbidities at the peripheral, spinal, and supraspinal levels. Currently, small molecule drugs targeting epigenetic machinery function globally, modifying the genome across multiple cells and tissues. Appropriate targeting of epigenetic therapeutics is needed to enhance specificity and reduce systemic side-effects. This targeting can be tissue-, cell- or gene-specific. Although this statement reflects common wisdom that good agents should be “magic bullets” it isn’t clear that “dirty” drugs will not have a therapeutic value. It is possible that general DNA methylation inhibitors such as RG108 (Machnes et al., 2013) or methyl donors such as SAM (Fuso et al., 2011) or general HDAC inhibitors such as SAHA (Hahnen et al., 2006) would be effective in treating chronic pain with the appropriate dosing and scheduling. Moreover, since DNA methylation associated with chronic pain appear to be widespread, it is possible that general modulators of DNA methylation that act system-wide will be required.

Although gene-targeting DNA methylation modifiers are still only a hypothetical approach, of potential therapeutic value might be gene-specific epigenetic targeting through recently developed genome editing technology. For example, TET-TALE fusion proteins have been shown to effectively target and demethylate individual genes *in vitro* (Maeder et al., 2013) and the recent development of Cas9 systems offer novel and flexible approaches to individual gene targeting (Hsu et al., 2014). While these approaches offer ~20–100 bp resolution, their development remains in its infancy and they have only been developed as tools for research.

The therapeutic interventions currently thought to offer some benefits through DNA methylation exist include nutritional inputs such as folate and other methyl donor intermediates. Relevant to neuropathy, folate has been shown to aide axonal regeneration in a dose-dependent manner through DNA methylation machinery and folate metabolism (Iskandar et al., 2010). Similarly, other methyl donors such as dietary choline are critical for fetal hippocampal brain development via genomic and gene-specific DNA methylation patterns (Niculescu et al., 2006).

## Concluding Remarks

Despite its high prevalence, little is known about the brain mechanisms underlying chronic pain and its associated co-morbidities. This hypothesis paper builds upon previous publications where we propose that DNA methylation contributes to the chronic changes in behavior and gene expression in neuropathic mice. Furthermore, it provides proof of principle evidence for linking peripheral injury-triggered central changes in DNA methylation and transcriptional regulation. Finally, we propose that long-term alterations in DNA methylation could provide a molecular substrate for chronic pain-related changes in the CNS, forming a “memory trace” for pain in the brain.

## Conflict of Interest Statement

The authors declare that the research was conducted in the absence of any commercial or financial relationships that could be construed as a potential conflict of interest.
